# Building the parasitic wasp *Encarsia*: a tale of three tails

**DOI:** 10.3389/finsc.2026.1861915

**Published:** 2026-09-16

**Authors:** Francesco Nugnes, Antonio P. Garonna, Paolo A. Pedata, Donato Mancini

**Affiliations:** 1 Institute for Sustainable Plant Protection - National Research Council (IPSP-CNR), Portici, Italy; 2 Department of Agriculture, University of Naples “Federico II”, Portici, Italy

**Keywords:** cleavage pattern, embryogenesis, *Encarsia lutea*, evolution of development, extra-embryonic membranes, parasitoid wasps, teratocytes

## Abstract

Embryonic development in hymenopteran parasitoids exhibits remarkable diversity, often associated with shifts in life history strategies such as the transition from ecto- to endoparasitoidism. In particular, reduction or loss of yolk has been linked to profound modifications of early developmental processes, including cleavage patterns and the formation of extra-embryonic membranes (EEMs). In this study, the embryogenesis of *Encarsia lutea* (Hymenoptera: Aphelinidae), a common whitefly parasitoid, was investigated using light and fluorescence microscopy. Eggs and developing embryos were collected at defined time intervals following oviposition, stained for F-actin and nuclei, and analyzed to characterize cleavage dynamics, blastoderm formation and the origin of extra-embryonic membranes. Observations were interpreted in a comparative framework with previously described congeneric species. The egg of *E. lutea* is oligolecithal and exhibits hydropic behavior requiring the uptake of fluids from the host haemocoel. Early development follows a predominantly peripheral cleavage pattern but is characterized by the precocious segregation of an apical blastomere at the eight-nuclei stage. Subsequently, a large portion of the blastoderm differentiate, giving rise to an extra-embryonic membrane that, together with the apical lineage, surrounds the embryo and later dissociates into numerous teratocytes. Comparative analysis reveals that *E. lutea* displays a combination of traits observed in two congeneric species, *Encarsia formosa* and *Encarsia pergandiella*, but follows a distinct developmental trajectory. In particular, the origin and formation of the EEM differ markedly among species, highlighting alternative solutions to similar functional constraints. These findings provide further evidence that early embryogenesis in parasitoid wasps is evolutionarily labile, even at low taxonomic levels, and suggest that developmental diversification within the genus *Encarsia* is shaped by a complex interplay between nutritional strategies, life history, and genetic flexibility.

## 1 Introduction

Developmental programs in animals have long been considered broadly conserved at the level of higher taxa and, as a consequence, alterations in early embryonic phases should be rare since, due to organismal constraints, their occurrence will cause mortality or alterations of adult phenotype. However, studies extending beyond traditional model species have revealed in many metazoans several examples of departures from the respective paradigmatic pattern taxa, with extreme divergence observed even among very closely related species (1, 2).

Shifts in life history strategies are commonly believed to be the primary force driving evolution of such modifications, which, interestingly, often occur without any respective changes in the adult body plan (3–5). Hymenopteran parasitoids are a notable group for which several examples of shift in early embryonic development have been observed since the beginning of the 20^th^ century (6–8). Parasitoid females lay their eggs on (ectoparasitoid) or inside (endoparasitoid) the host, which is finally killed by the hatched larvae. Intimate interactions with hosts have directed the evolution of numerous adaptive strategies, particularly sophisticated, as life-style shifts to endoparasitoidism. Indeed, those species which develop in a hostile environment, are more likely to evolve specialized adaptations to evade host defenses and, in general, to challenge host physiology (9). 

A fascinating innovation, independently evolved in several lineages of endoparasitic wasps, is the production of eggs without yolk (alecithal) (10, 11). The development of this kind of egg differs markedly from the centrolecithal egg, the derived state for Insects, widely conserved throughout the different Orders, including the majority of Hymenoptera. In particular, in this latter group centrolecithal egg is adopted across a wide range of lifestyles, such as the phytophagous sawflies, the free-living Aculeata, and even most parasitoids, both ecto- or endoparasitoids. This kind of egg, rich in yolk and with a rigid chorion, develops with only minor variations from the canonical model described for *Drosophila*. The centrolecithal egg develops without swelling (anhydropic) through a superficial or peripheral cleavage in which nuclei resulting from multiple cleavage cycles migrate toward the periphery of the egg forming a syncytial blastoderm that successively cellularizes (12, 13). In contrast, alecithal egg, which remarkably swells during the first phases (hydropic) (14, 15), undergoes holoblastic (total) division. Alecithal eggs are considered a preadaptation for other developmental shifts, such as the production of specialized extra‐embryonic membranes (EEMs), and, as an extreme consequence, polyembryony (5, 16).

Extra‐embryonic membranes are special structures enveloping the embryo during its development (17) that can have very different embryonic origin (18). EEMs exert nutritional, defensive and regulative roles on host physiology (19), and in some case extend their function after hatching, dissociating into special cells, the teratocytes, freely floating in the host haemolymph (20). Teratocytes, such as EEMs, are involved in larval nutrient uptake (21–27), host hormonal regulation (28–34) and host immune suppression (35–40), through the secretion of specific proteins. Recently, proteotranscriptomic analysis of teratocytes of the braconid *Cotesia flavipes* Cameron showed the production and release in the host haemolymph of up to 57 polypeptides (41). During larval development teratocytes undergo profound cytological changes, often considerably increasing both in diameter (42, 43) and ploidy level (42, 44, 45), even if in some systems they do not vary in size (46). Teratocytes in general do not undergo cellular division (20, 47–49), with the interesting exception of the aphelinid *Encarsia pergandiella* Howard whose larger polynucleated teratocytes are able to divide, thus progressively reducing their size (50).

*Encarsia* Foerster (Hymenoptera: Aphelinidae) is a speciose genus of tiny endoparasitic wasps, comprising 486 described species, which exhibit an interesting heteronomous ontogeny: indeed, females develop at expense of phytophagous insects, particularly Homoptera Aleyrodoidea and Coccoidea and, in much lesser extent, Aphidoidea and eggs of Plataspidae, while males develop, with a few exceptions, as autoparasitoids (51). Even though several species of *Encarsia*, including the iconic *Encarsia formosa* Gahan possess a centrolecithal egg (52, 53), other species have exploited an alternative reproductive strategy based on alecithal egg, as reported for *Encarsia berlesei* (Howard) and *Encarsia citrina* (Craw) (45) and *Encarsia pergandiella* Howard (53).

In this framework, the embryonic development of *Encarsia lutea* (Masi), a common whitefly parasitoid, was investigated and discussed in a comparative context with other species of the genus *Encarsia*.

## 2 Materials and methods

### 2.1 Insect cultures

*Encarsia lutea* is a solitary, arrenothokous endoparasitoid of several species of whiteflies, including economically important species, such as *Trialeurodes vaporariorum* (Westwood) (Hemiptera: Aleyrodidae) and *Bemisia tabaci* Gennadius (Hemiptera: Aleyrodidae). Laboratory cultures of both the parasitoid and its natural host, *T. vaporariorum*, were started from material collected in several sites of Campania (Italy). *Trialeurodes vaporariorum* was reared on bean plants *Phaseolus vulgaris* L. cv. ‘Borlotto nano’ (Fabaceae) in 10 cm diameter pots in an environmental chamber at 25 ± 1 °C, 60–70% r.h. and LD 12:12. Experimental infestations of *T. vaporariorum* of uniform age class were obtained by exposing new bean plants to ovipositing whiteflies for a period of 3–4 days. *Encarsia lutea* was reared under the same environmental conditions of its host. Since this species is a facultative autoparasitoid, *i.e.* male larvae, hatched from unfertilized eggs, develop at expense of female larvae of its own or other *Encarsia* species (51), the two sexes were reared according to their specific trophic requirements. In more details, females were obtained by exposing third and fourth nymphal stages (N_3_–N_4_) of whiteflies to mated *E. lutea* females, while males were obtained by exposing whitefly pupae parasitized by *E. formosa* (late pre-imaginal stages) to virgin *E. lutea* females.

### 2.2 Ovaries of *Encarsia lutea*

Ovaries were extracted with micropins from adult females in Ringer solution (NaCl 0.80 g; CaCl_2_ 0.02 g; KCl 0.02 g; NaHCO_3_ 0.02 g, in 100 ml of distilled water, pH 7.2) and fixed in formaldehyde saline (40% formaldehyde 10 ml, NaCl 0.9 g in 90 ml of distilled water) for 1–2 h. Ovaries, after rinsing in Ringer for 10’, were stained in acetic carmine and rinsed in dilute acetic acid (distilled water 50 ml, 50% acetic acid 50 ml) to remove the excess of stain. Finally, ovaries were slide mounted in Ringer solution mixed with a drop of glycerol and photographed with Leica Imaging Workstation Q5501W (Wetzlar, Germany).

### 2.3 Embryology of *Encarsia lutea*

Oviposition arenas were obtained by laying a leaf disk, infested by N_3_–N_4_ whiteflies, into a Petri dish (35 mm diameter) filled with a thin layer of 5% agar solution. Arenas were exposed to ten 24 h-old mated females for about 5 min and whiteflies were tagged with a fine-tip marker as they were parasitized. Parasitoids were removed and arenas were incubated under rearing conditions.

Samples for the analysis of cytological events during embryogenesis were obtained by dissection of marked hosts at variable intervals (between 5 min and 90h after deposition) in Phosphate buffered saline (PBS) (NaCl 8.00 g, KCl 0.20 g, Na_2_HPO_4_ 1.44 g, KH_2_PO_4_ 0.24 g, in 100 ml of distilled water, pH 7.4).

Embryos, collected by dissection of the host body, were transferred onto glass slides and fixed overnight in 10% formaldehyde (corresponding to 4% paraformaldehyde) in PBS in a humid chamber at 4 °C. After fixation, samples were rinsed in PBS 2–3 times for 3 min each, and then permeabilized in 0.3% PBS/Triton X-100 for 5 min. To remove residual formaldehyde and Triton, a further rinse in PBS for 3 min was performed.

Fixed embryos were prepared for fluorescence microscopy in a two-step staining procedure to visualize F-actin filaments (for indirect evidence of cellular boundaries) and nuclei. F-actin was stained by incubation in Rhodamine–Phalloidin (Sigma-Aldrich, Milan, Italy) at a concentration of 1 μg/ml (1 μl applied per sample) for 20 min at 4 °C in a humid chamber. Following a rinse in PBS, nuclei were stained by incubation in Hoechst 33258 (Sigma-Aldrich, Milan, Italy) at 1 μg/ml for 5 min at 4 °C in a humid chamber. After a final rinse in PBS to remove excess staining solution, samples were slide-mounted in a drop of glycerol (90%) with n-propyl gallate (2.5%) (54). Mounted samples were stored at 4 °C in a humid chamber and kept strictly in the dark to avoid photodegradation of the fluorochromes. Transfers between solutions were carried out using microcapillaries (Microcaps, Sigma-Aldrich, Milan, Italy) fitted with tips of 60-200 μm diameter.

Samples were observed with a Zeiss Axiophot 2 microscope (Göttingen, Germany) equipped with rhodamine–fluorescein and UV filter sets. Images were captured using a Zeiss AxioCam HRC digital camera (Oberkochen, Germany). Different focal planes were acquired and Z-stacked; the resulting images from the two fluorescence channels were merged using ImageJ software (55).

### 2.4 Observation of larval stages and teratocytes

To observe larval development and teratocyte dynamics, parasitized pupae of *T. vaporariorum* were dissected in PBS from egg hatching at approximately 24 h intervals until the prepupal stage.

Samples were subsequently fixed in saline formaldehyde for approximately 2 h to facilitate subsequent handling and observation. For each larval stage, teratocytes were counted and their diameters were measured. To evaluate morphometric changes of teratocytes during larval growth, for each larval stage a subsample was collected from several parasitized hosts, performing a Welch’s ANOVA (to account for heteroscedasticty) followed by Games-Howell pairwise comparisons. Statistical analyses were performed in R (version 4.5.1) (56) using the packages readxl, dplyr, ggplot2, car, and rstatix. Due to inherent difficulties in recovering all teratocytes from parasitized hosts only an indicative value of the number of teratocytes is provided for the three larval stages of *E. lutea*.

## 3 Results

### 3.1 Ovaries of *Encarsia lutea*

*Encarsia lutea* showed two typical meroistic polytrophic ovaries, each one consisting of three ovarioles, with oocytes at progressive maturation stages surmounted by large nurse cells and surrounded by follicle cells (**Figure 1A**).

**Figure 1:**
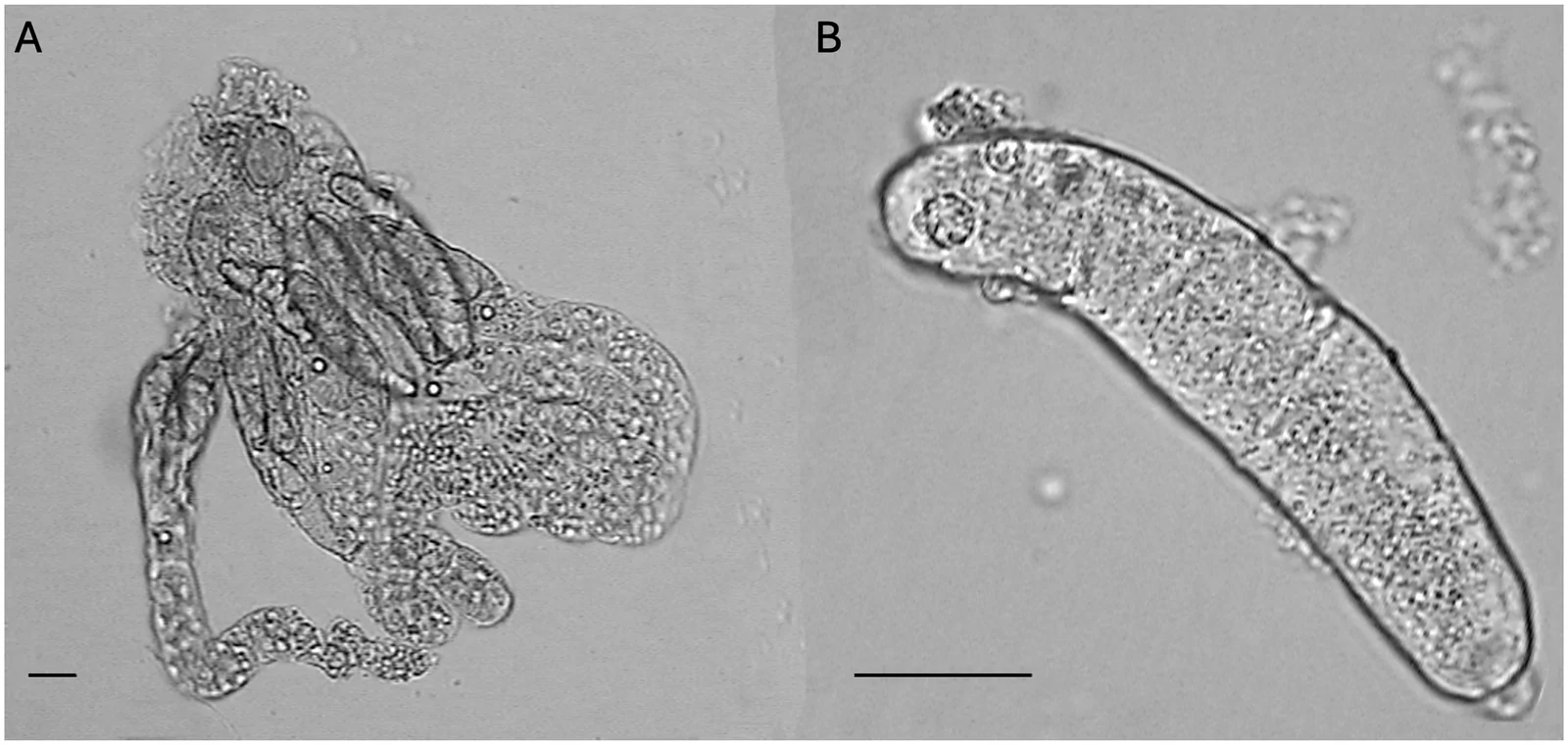
Ovaries **(A)** and freshly laid egg **(B)** of *Encarsia lutea* (bar 20µm).

The freshly laid egg of *E. lutea* (**Figure 1B**), resembling the ovarian egg, was of oligolecithal type, *i.e.* with a reduced yolk content. It presented an elongated shape and had no stalk. At deposition it measures 88.3 µm (± 4.10 µm) x 23.7 µm (± 1.99 µm) (n=15), but a few minutes after deposition, the egg showed a hydropic behavior, gradually increasing in volume by absorbing fluids from the host.

### 3.2 Embryology of *Encarsia lutea*

A schematic overview of the timing and sequence of the main embryological events is provided in **Supplementary Figure 1**. During the first hour the sperm penetrated in the egg (first order oocyte stage) (**Figure 2A**) and lost its tail while the nucleus started the first division with the formation of the second order oocyte and of the first polar body (PB) (**Figures 2B, C**). At the end of the 2nd maturation division, the male and female pronuclei, both spherical in shape and with a low-density appearance, were in close proximity, while the 3 PBs were located at the egg periphery in a subpolar position (**Figure 2D**). Between the 2nd and 3rd hour, the two pronuclei fused forming the synkaryon, while the PBs appeared very dense and bright, probably as a result of polyploidization events (**Figures 2E–G**). Between the 3rd and the 4th hour, the synkaryon divided producing the first two cleavage nuclei (**Figure 2H**).

**Figure 2:**
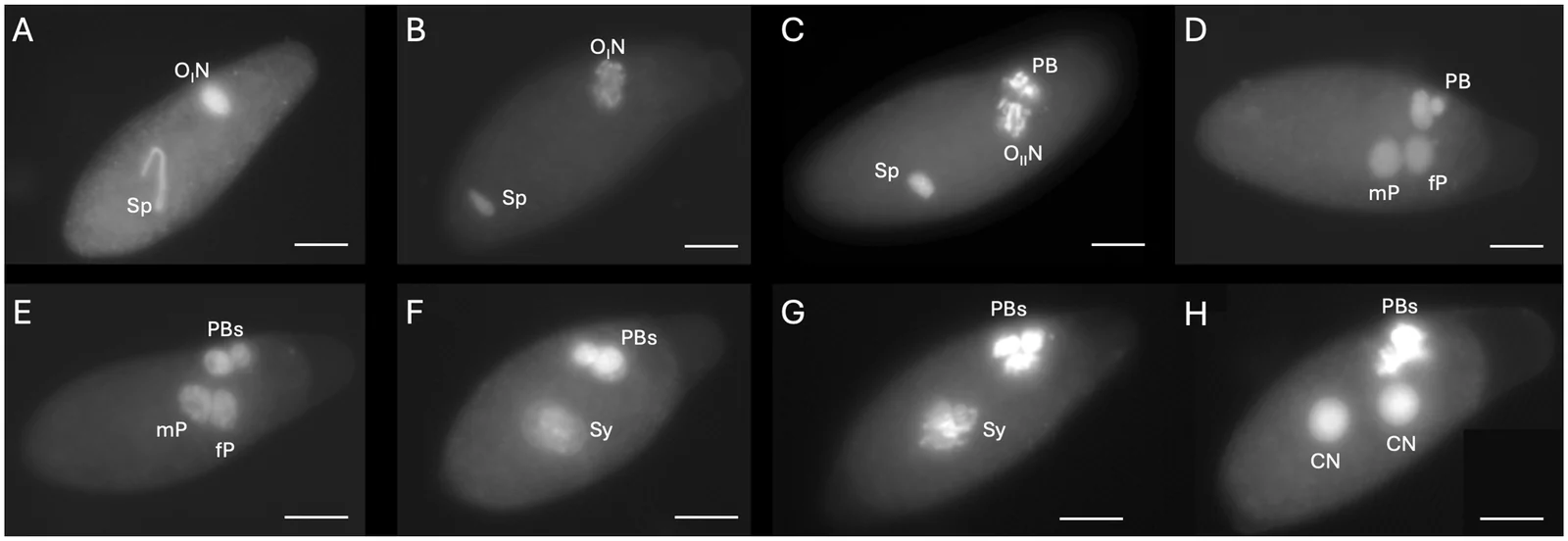
**(A)** Sperm (Sp) penetrates in the egg with the first order oocyte nucleus (O_I_N); **(B)** Sp loses its tail and the O_I_N starts the first division; **(C)** the resulting nucleus of the second order oocyte (O_II_N) and first Polar Body (PB) prepare for the second division; **(D)** male (mP) and female pronuclei (fP) and three PBs; **(E)** mP and fP approaching each other; **(F)** pronuclei fuse to form the synkaryon (Sy); **(G)** Sy starts the first cleavage; **(H)** first two cleavage nuclei (CN) with PBs (bar 20µm).

Successively, at the second cleavage, the polar bodies derivative (PD) appeared surrounded by a weak actin cortex (**Figure 3A**) and immediately after a similar cortex formed around one of the nuclei located at the apex of the embryo (**Figure 3B**). Successively, contraction of the actin cortex produced a constriction beneath the chorion and consequently the apical nucleus resulted confined in a separated blastomere, close to the PD (**Figure 3C**).

**Figure 3:**
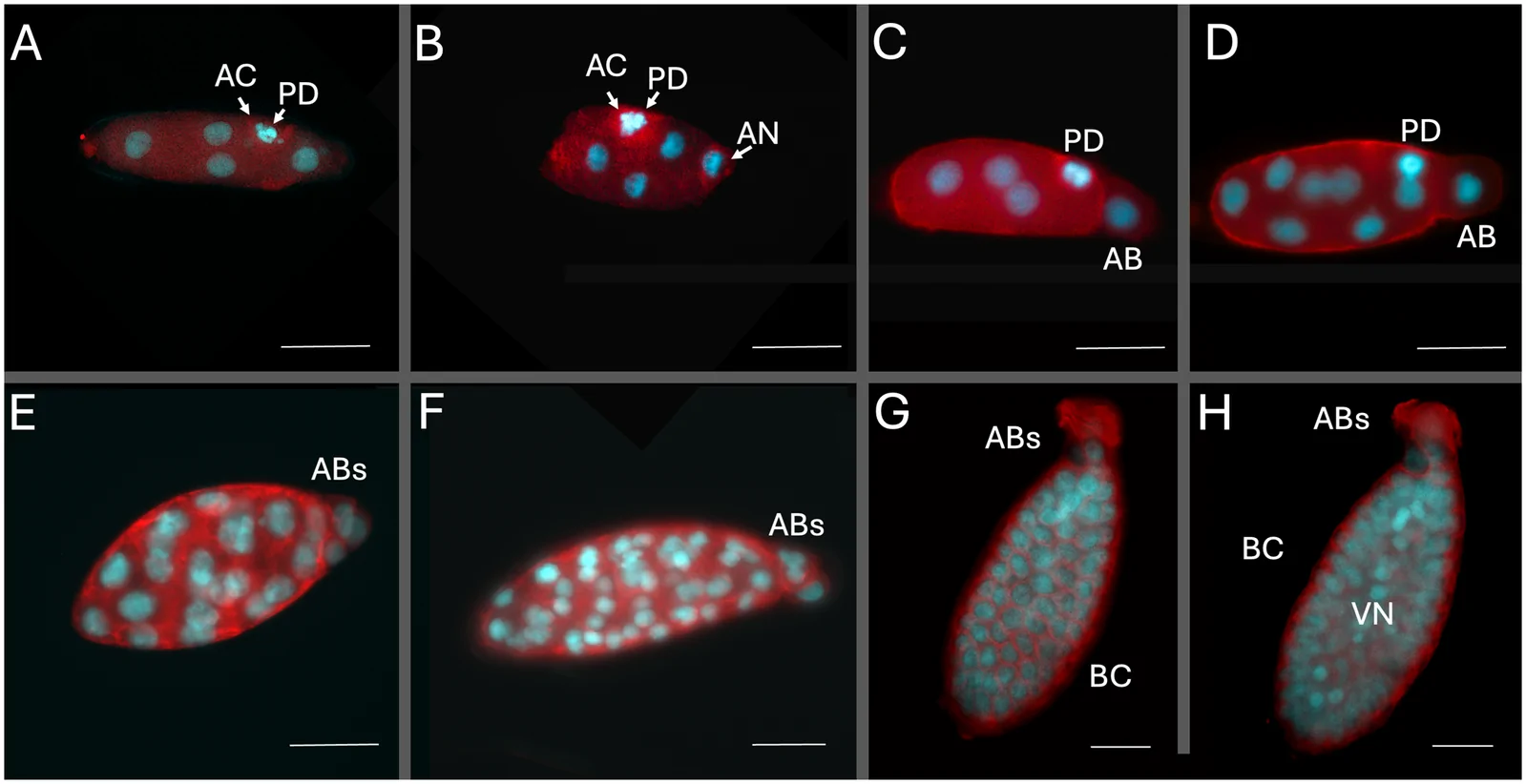
**(A)** Four cleavage nuclei phase with polar bodies derivative (PD) surrounded by an actin cortex (AC); **(B)** PD and apical nucleus (AN) individually surrounded by AC; **(C)** the AN completely segregates in an apical blastomere (AB); **(D)** 8 cleavage nuclei phase with PD no longer surrounded by AC; **(E)** embryo at the 5^th^division and **(F)** at the 6^th^ division: the AB has divided producing four ABs; **(G)** Superficial and **(H)** internal view of the blastodermic phase with blastodermal cells (BC) at the periphery and vitelline nuclei (VN) distributed in the center of the egg (bar 20µm).

Around the 5^th^ hour, at the third cleavage, the PD no longer appeared surrounded by the actin cortex (**Figure 3D**). Between the 6^th^ and the 7^th^ hours, at the fifth cleavage, the embryo reached the 32-nucleus phase, with 28 nuclei in its largest portion, while the apical blastomere produced by parallel division 4 nuclei roughly delimited by actin boundaries. In this phase, the PD was no longer detectable (**Figure 3E**). During the next hours, embryonic nuclei underwent several cleavage stages, except for the four presents in the apical cap, which still appeared delimited by actin boundaries (**Figure 3F**). After the 20^th^ hour most nuclei migrated towards the periphery to form a blastoderm, with several vitellophages persisting in the central yolky region, while the apical cap remained well separated (**Figures 3G, H**).

Around the 24^th^ hour, following further cell divisions, the chorion stretched and the constriction disappeared, while the apical cells moved closer to one another and to the blastoderm cells. At the same time, numerous cells of the ventral region of the blastoderm assumed a columnar shape, thus suggesting a forthcoming transverse division (**Figure 4A**).

**Figure 4:**
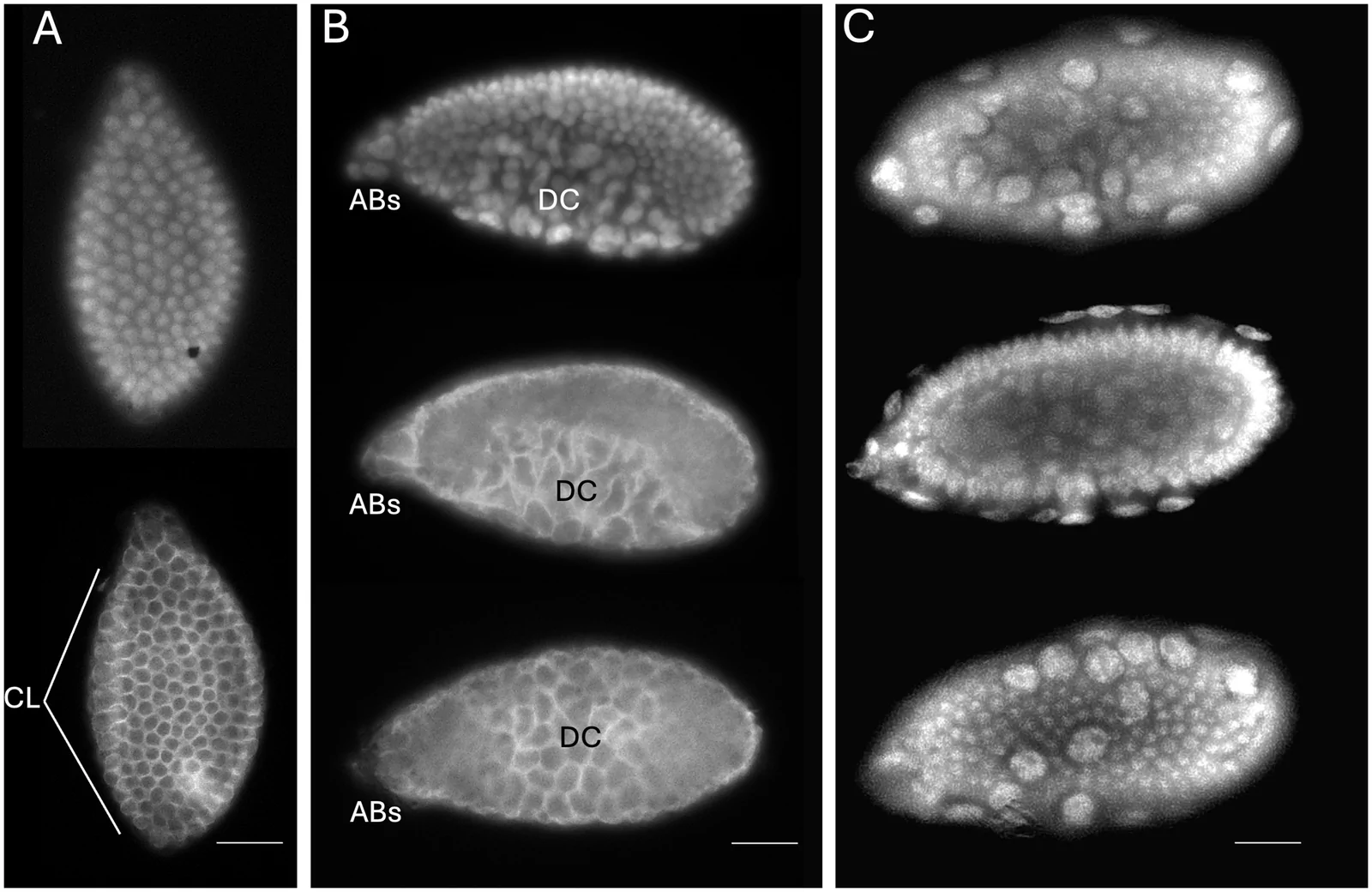
**(A)** Nuclei (top) and cellular boundaries (bottom) of the blastodermal cells, showing the formation of a columnar layer **(CL)** in the ventral region; **(B)** Blastoderm “delamination” cells (DC): nuclei (top), cellular boundaries in lateral (middle) and ventral (bottom) view; apical blastomeres (ABs) still separated from the blastoderm; **(C)** Different focal planes of the same egg showing DC surrounding the blastoderm (bar 20µm).

Between the 28^th^ and 29^th^ hours, the columnar cells gave rise to a layer of about 50–60 large cells, presenting very large nuclei and an irregular profile of the membrane, overlaying a broad region of the blastoderm, whose cells appeared smaller and more tightly packed (**Figure 4B**). Around the 32^nd^-33^rd^ hours these large cells distributed all over the surface of the blastoderm under the chorion (**Figure 4C**). At about 70–80 hours the embryo was in an advanced stage of development, and the surrounding cells appeared as a stretched layer, forming an extra-embryonic membrane (EEM) (**Figure 5A**) that at hatching, occurring at about 90 h, appeared still tightly compacted around the larva (**Figure 5B**). Shortly after, the EEM dissociated into 50–60 teratocytes, very regular in shape and dimension, showing a mean diameter of 22.8 ± 1.28 μm (n=250) (**Figure 5C**). During larval development, while their number reduced, their size increased. Indeed, during the second larval stage, the mean number of teratocytes was approximately 35 with a mean diameter of 27.9 ± 2.88 μm (n=150) and during the mature larval stage, teratocyte number was less than 30 and their mean size was 37.9 ± 4.91 μm (n=200). The number of teratocytes differed significantly among the three groups (Welch’s ANOVA: F _(2, 264.54)_ = 1045.78, p < 0.001). Games–Howell *post hoc* comparisons showed that all pairwise comparisons were significantly different (all p < 0.001).

**Figure 5:**
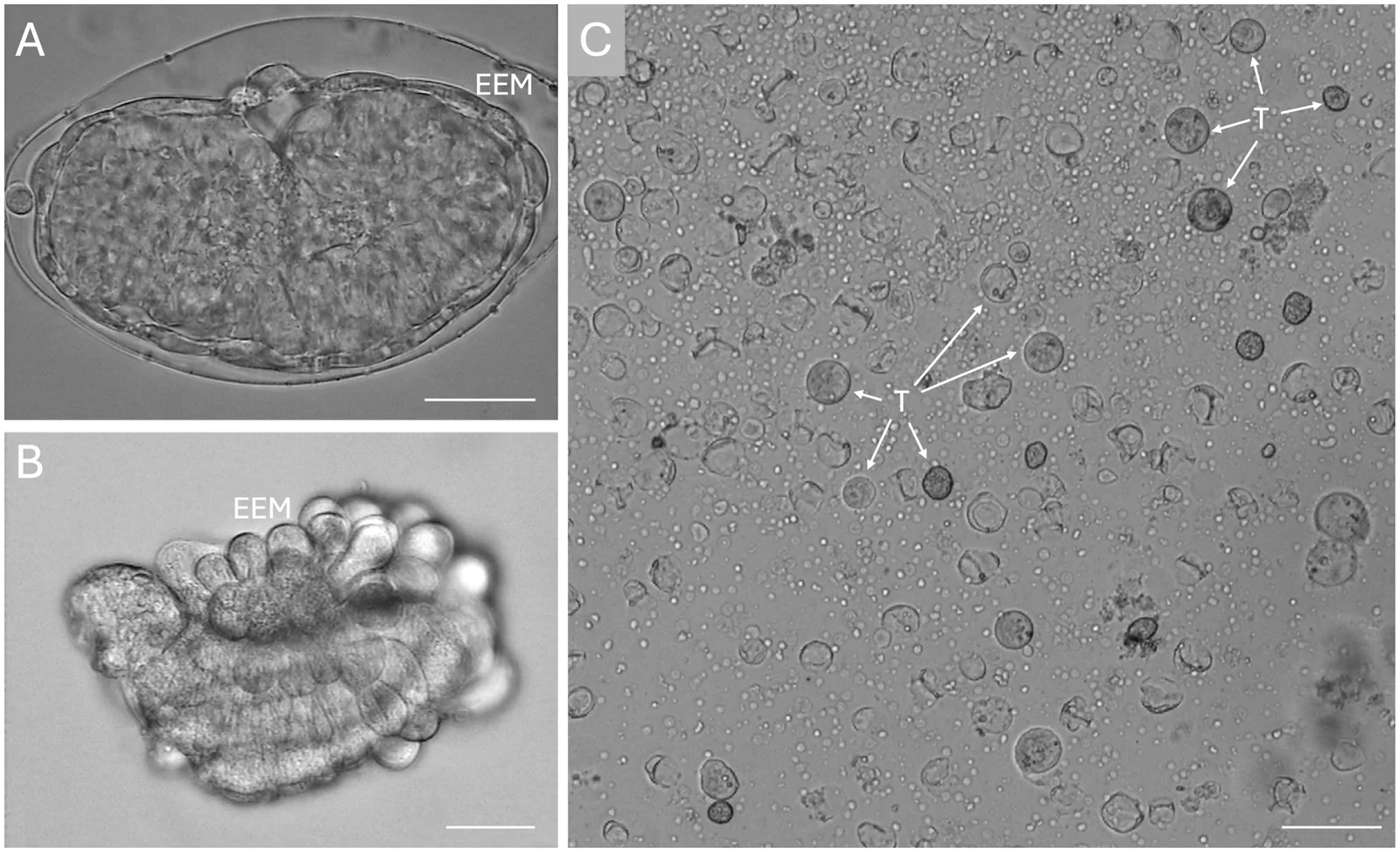
**(A)** extra-embryonic membrane (EEM) stretched all over the embryo in an advanced developmental phase; **(B)** freshly hatched larva still surrounded by undissociated EEM; **(C)** teratocytes (T) (bar 50µm).

## 4 Discussion

Embryonic development of *E. lutea* started from an oligolecithal (moderate amount of yolk) egg, which required the absorption of fluids and nutrients from the host haemocoel, thus rapidly starting to swell after oviposition (hydropic behavior). The subsequent phases of embryogenesis followed an almost standard peripheral division pattern, with the exception of a precocious segregation of an apical blastomere at the 8-nucleus stage, whose division products remained well separated from the remainder of the embryo. Later on, but before the formation of the germ band, a wide portion of the blastoderm produced a layer of large cells with giant nuclei, likely polyploid. These cells in a more advanced phase, together with the apical blastomeres, completely surrounded the developing embryo as a continuous EEM. This membrane subsequently dissociated at hatching into 50–60 teratocytes that freely floated in the host haemocoel.

Interestingly the embryonic development of *E. lutea* showed features that are intermediate between the two congeneric species, *E. formosa* and *E. pergandiella*, whose embryology has been thoroughly investigated (53). The interspecific differences in the developmental events are mainly linked to differences in nutritional strategies at the ovarian level. Indeed, although all these species possess meroistic polytrophic ovaries, typical of holometabolous insects, they differ in the amount of yolk present in their eggs. Differences in yolk content are clearly reflected in the egg size of the three species with oligolecithal egg of *E. lutea* occupying an intermediate position between the centrolecithal egg of *E. formosa* and the alecithal egg of *E. pergandiella* (53) (**Table 1**). The absence of yolk in *E. pergandiella* egg is further supported by the failure to detect the vitellogenin gene, which is instead present in *E. formosa* (57).

**Table 1:** Comparative overview of egg type and developmental traits in three *Encarsia* species.

Trait		*E. pergandiella*	*E. lutea*	*E. formosa*
Freshly laid egg measures in µm (± s.d.)	L	74.1 (4.44)	88.3 (4.10)	120.2 (6.44)
	W	18.5 (1.06)	23.7 (1.99)	40.5 (1.97)
Centrolecithal egg		〇	〇	X
Oligolecithal egg		〇	X	〇
Alecithal egg		X	〇	〇
Anhydropic egg		〇	〇	X
Hydropic egg		X	X	〇
Holoblastic cleavage		X	〇	〇
Superficial cleavage		〇	X	X
Extra embryonic membrane		X	X	〇
Teratocytes		X	X	〇
number; size (in µm) at hatching		10; 55-85	50-60; 20-25	n.p.

Absence (〇) or presence (X) of a specific trait, s.d., standard deviation; n.p., not present.

Regarding the first embryonic phases, while in *E. formosa* the centrolecithal anhydropic egg undergoes canonical peripheral cleavage, in *E. pergandiella* the alecithal hydropic egg, except for a single apical blastomere segregating at the 8-nucleus stage, follows a holoblastic (total) cleavage pattern with complete compartmentalization between the third and fourth divisions. From this stage onward the apical blastomere, two posterior cleavage nuclei and two other cleavage nuclei associated with the polar bodies derivative (PDs) are recruited to give rise to an EEM that completely surrounds the developing embryo (53).

In synthesis, even though the oligolecithal egg of *E. lutea* underwent peripheral cleavage as in *E. formosa*, its reduced yolk content required the absorption of fluids and nutrients from the host, resulting in hydropic behavior, similar to that observed in *E. pergandiella* (**Table 1**). Another trait shared with the latter species is the development of an EEM, which is noteworthy since such envelopes are generally associated with yolkless eggs. However despite these similarities, the embryonic development of *E. lutea* represents a distinct evolutionary novelty. Indeed, even if the EEMs of both species have a double origin, the cytological events leading to their formation are profoundly divergent, except for the precociously segregating blastomere at the 8-nucleus stage. In *E*. *pergandiella*, the regions of the EEM, already defined at the end of the fourth division, are formed through the successive recruitment of several cleavage nuclei, some autonomously and others associated with the PDs, whereas in *E. lutea*, with the exception of the apical blastomere, most EEM nuclei originated later from the cellularized blastoderm. Moreover, no evidence was found for a role of PDs in EEM formation in *E. lutea*.

The EEM of *E. lutea* cannot be easily assigned to any of the types described in parasitic Hymenoptera (18). According to the classification proposed by these authors, the true serosa derives from the extraembryonic cells of the blastoderm, which successively proliferate over the embryo; the pseudoserosa derives from a simultaneous process of delamination of the still undifferentiated blastoderm; and the deutoserosa originates from internal cells initially associated with the yolk, which, as the blastoderm expands, are displaced to the surface and proliferate thus originating the EEM. These three EEMs have in common the fact they are genetically identical to the embryo, since they derive exclusively from cleavage nuclei. A completely different type of EEM is represented by the trophamnion, which derives from the PDs, alone or associated with one or more cleavage nuclei. As already mentioned, EEM of *E. lutea* presented a double origin.The chronological sequence of embryological events, including the localized thickening of the blastoderm, the successive synchronized differentiation of cells with giant (presumably polyploid) nuclei and the marked remodeling of their actin cytoskeleton, is suggestive of a process of simultaneous delamination. Indeed, the separation of cell layers could trigger a developmental switch, promoting the transition from mitotic divisions to rapid endoreplication cycles, a classic hallmark of extraembryonic cell lineage determination in insects (58). If this should be the case, the portion of the EEM derived from this area of the blastoderm, should be classified as a pseudoserosa. On the contrary, the few cells deriving from the unique precociously segregated blastomere do not clearly match any of the described categories. In *E. pergandiella*, the portion of the EEM derived from blastomeres at both apices has been tentatively considered as a serosa-like membrane, even in the absence of a blastoderm, while the portion originated from PDs in association with cleavage nuclei can be clearly attributed to a trophamnion (53). Comparison between *E. lutea* and *E. pergandiella* highlights that the cytological events leading to the 8-nucleus stage, including the compartmentalized apical blastomere, are strikingly similar. However, due to the subsequent formation of a blastoderm in *E. lutea*, the definition of “serosa-like” does not appear appropriate for the apical portion of its EEM. Understanding the homology of these cytological events in an evolutionary framework will require identification and comparative analysis of morphogenetic determinants underlying cell fate specification. At present, it can only be noted that in both species, from the earliest stages, PDs appear anchored by an actin cortex to the border of the apical region where segregation of an early blastomere occurs. Whether this process is orchestrated by the PDs themselves remains an intriguing hypothesis that would require further investigation. Interestingly, a role for polar bodies in specifying embryonic cell fate has recently been demonstrated in a mammalian model (59).

Another difference between *E. lutea* and *E. pergandiella* concerns the number and size of teratocytes derived from dissociation of the EEM. In *E. lutea*, teratocytes at hatching were more numerous, smaller, and more regularly shaped compared with *E. pergandiella*, in which EEM dissociation produces on average about 10 larger and irregular teratocytes, with a diameter ranging between 22 and 114 µm (most of them in the range 50-85 µm) (50). Moreover, during larval development in *E. lutea*, teratocyte number progressively decreased in parallel with their increase in size, as reported for other host-parasitoid associations, where teratocyte number is progressively reduced by larval ingestion (60), degeneration, or host expulsion (61). This pattern differs from *E. pergandiella*, in which mean number of teratocytes initially increases while their size decreases, due to the division of larger and polynucleated teratocytes into smaller ones. Larger and less numerous teratocytes are also found in two other congeneric species possessing an alecithal egg, such as *Encarsia berlesei*, with about 20 teratocytes measuring 60-100 µm, whose ploidy level greatly increases during larval development, and *Encarsia citrina*, with only five teratocytes of about 250 µm (45). Overall, teratocyte number appears to be highly species-specific, as reported for several other systems, ranging from as few as five in *E. citrina* (45) to more than 900 in the braconid *Microplitis croceips* (Cresson) (62).

Embryonic development of *E.* *lutea* provides further evidence that early embryogenesis is not phylogenetically conserved among hymenopteran parasitoids. However, it should be emphasized that remarkable diversity of developmental patterns found within the genus *Encarsia* is unusual at this taxonomic level, as a comparable diversity within Hymenoptera can be observed only at the family level, such as in the Braconidae (5, 17).

The transition from ecto- to endo-parasitoidism has been proposed as a major driving force shaping the evolution of developmental strategies in Hymenoptera, prevailing over constraints imposed by ancestry (5, 63). Apparently, evolution of alternative developmental strategies in hymenopteran parasitoids appears to be strictly associated with a reduction in yolk content, due to the loss of the gene of vitellogenin (or in alternative of its function), the precursor of yolk proteins, an event that has occurred independently in at least five nonrelated lineages (11). Endoparasitoidism may have been an important factor relaxing selective pressure on genes involved in yolk production, since the embryo develops in the host haemolymph from which it can absorb nutrients, such as amino acids (64, 65). However, Zhao et al. (11) suggested that endoparasitism alone is not sufficient to explain the loss of vitellogenin, as their genomic analysis showed that about 35% of examined endoparasitoids retain one or more copies of the genes. A similar pattern is observed within the genus *Encarsia*, where a preliminary survey indicates that centrolecithal eggs are still widely distributed among species (45).

A further complication in clarifying this picture lies in the limited understanding of whether developmental shifts occur abruptly, as seems to be the case in some sea urchin species (1), or whether they proceed more gradually. In the genus *Encarsia*, developmental strategies adopted by some species suggest the emergence of the latter scenario, with some species representing transitional conditions toward the complete loss of vitellogenin function, as *E. lutea*, which presents an oligolecithal egg with intermediate features between the centrolecithal egg of *E. formosa* and the alecithal egg of *E. pergandiella*.

Thus, the evolution of alternative developmental strategies, beyond a simple lifestyle shift, is more likely driven by a complex interplay of phylogenetic history, environmental interactions, internal constraints, and genetic flexibility which together define the framework within which some developmental features are conserved while others can change. From this perspective, a high level of evolvability is expected when genetic traits are regulated by modular networks that can be reconnected into multiple, adaptive combinations (66). Nematodes, another phylum of Ecdysozoa, provide an elegant example as the high developmental plasticity observed in several clades originates by rearrangements of *Hox* genes, key regulators of body patterning in Bilateria (67).

The divergence of underlying genetic mechanisms and developmental pathways without a corresponding alteration of the final phenotype, known as ‘developmental system drift’ (DSD), (68), provides a conceptual counterpoint to the iterative deployment of a conserved genetic toolkit, reflected in the ‘deep homology’ of core developmental components (69, 70). While the latter highlights how deeply homologous genes can generate similar phenotypes across vast phylogenetic distances, DSD explains how phenotypic stasis can be maintained despite substantial regulatory reorganization, effectively decoupling the conservation of the ‘toolkit’ from the architecture of the developmental process. Thus, comparative analyses of developmental mechanisms in an EvoDevo framework require interpretation within a robust phylogenetic context in order to reconstruct character polarity (ancestral vs. derived), patterns of trait evolution (gain vs. loss), and instances of convergent evolution.

Interestingly, the holoblastic cleavage pattern observed in some highly derived endoparasitoids is reminiscent of the basal hexapods, Collembola and Protura, which exhibit this developmental mode (71, 72), thus considered the most likely ancestral condition in Hexapoda (73). In contrast, superficial cleavage has been repeatedly and independently acquired in several arthropod lineages that have diverged from the general holoblastic pattern (73). These bidirectional (“back-and-forth”) transitions between superficial and total cleavage represent a fascinating evolutionary puzzle, whose comprehension will require comparative data on the genetic mechanisms regulating embryogenesis across taxonomic levels, to understand whether similar developmental patterns originate through the reactivation of ancestral regulatory systems or through the evolution of distinct genetic pathways.

## 5 Conclusions

Even if in just over a century, embryologists have discovered a rather diversified range of developmental strategies in hymenopteran parasitoids, current knowledge captures only a fraction of the evolutionary transitions in embryogenesis of this group. The discovery of a high embryonic diversification in a single genus represents a further *caveat* about generalization inferred from a few selected model systems, erroneously considered representative of a whole group, which could obscure the existence of different evolutionary trajectories. On the other hand, occurrence of extreme developmental divergence makes the megadiverse genus *Encarsia* an ideal microevolutionary model to test hypothesis about how significant shifts in selective pressures have shaped the differential evolution of alternative embryonic patterns in closely related species.

Modern genomic techniques, such as advanced sequencing, transcriptomics, and gene silencing will represent a powerful tool to analyze gene regulatory networks and identify specific genetic changes responsible for developmental divergence, offering a deeper understanding of how natural selection shapes development and adaptation.

## Data Availability

The original contributions presented in the study are publicly available. The raw data supporting the findings of this study can be found in the following GitHub repository: https://github.com/FrankNug/En-lutea.
