# Supplementary material for: Building the parasitic wasp Encarsia: a tale of three tails
Source: Front Insect Sci. 2026 Sep 16;6:1861915. doi: 10.3389/finsc.2026.1861915 (PMC13624162; doi:10.3389/finsc.2026.1861915)
Supplement: Supplementary file 1 [file DataSheet1.pdf]

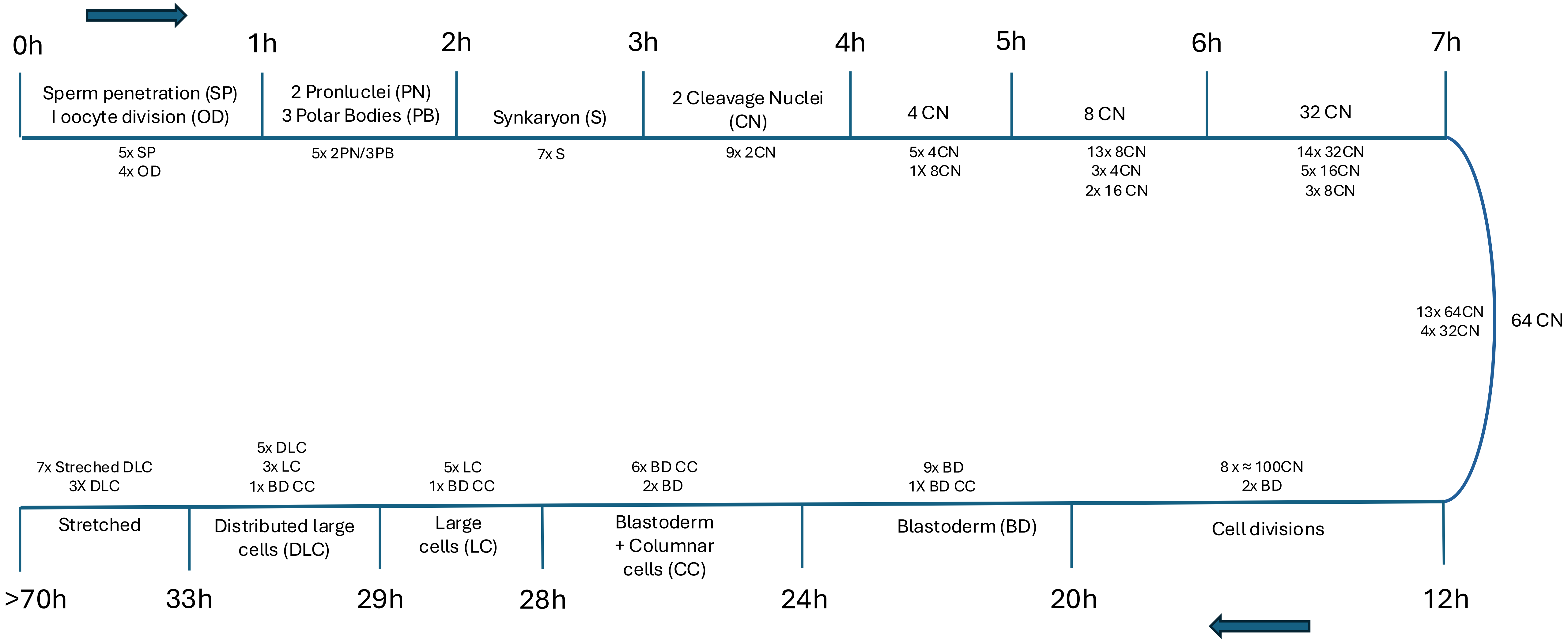
0h
1h
2h
3h
4h
5h
6h
7h
Sperm penetration (SP)
I oocyte division (OD)
2 Pronluclei (PN)
3 Polar Bodies (PB)
Synkaryon (S)
2 Cleavage Nuclei (CN)
4 CN
8 CN
32 CN
5x SP
4x OD
5x 2PN/3PB
7x S
9x 2CN
5x 4CN
1X 8CN
13x 8CN
3x 4CN
2x 16 CN
14x 32CN
5x 16CN
3x 8CN
13x 64CN
4x 32CN
64 CN
7x Streched DLC
3X DLC
5x DLC
3x LC
1x BD CC
5x LC
1x BD CC
6x BD CC
2x BD
9x BD
1X BD CC
8 x ≈ 100CN
2x BD
Stretched
Distributed large cells (DLC)
Large cells (LC)
Blastoderm + Columnar cells (CC)
Blastoderm (BD)
Cell divisions
>70h
33h
29h
28h
24h
20h
12h
